# The effect of low-dose and high-dose low-molecular-weight-heparin and aspirin thromboprophylaxis on clinical outcome and mortality in critical ill patients with COVID-19: A retrospective cohort study

**DOI:** 10.15537/smj.2022.43.7.20220038

**Published:** 2022-07

**Authors:** Ali Eman, Onur Balaban, Kezban Ö. Süner, Yaşar Cırdı, Fatih Şahin, Gürkan Demir, Özge Pekşen, Ahmet Musmul, Ali F. Erdem

**Affiliations:** *From the Department of Anesthesiology and Reanimation (Eman, Balaban, Şahin, Demir, Pekşen, Erdem); from the Department of Intensive Care (Süner), Sakarya Training and Research Hospital; from the Department of Intensive Care (Cırdı), faculty of Medicine, Sakarya University, Sakarya; and from the Department of Medical Services and Techniques (Musmul), Eskisehir Osmangazi University Vocational School of Health Services, Eskisehir, Turkey*.

**Keywords:** COVID-19, critical illness, anticoagulants, heparin, aspirin

## Abstract

**Objectives:**

To assess the effect of different thromboprophylaxis regimens on clinical outcomes and mortality of critical ill patients with coronavirus disease -19 (COVID-19).

**Methods:**

We investigated the medical records of patients with positive COVID-19 (using polymerase chain reaction test) who were admitted to the intensive care unit (ICU) at Sakarya University Hospital, Sakarya, Turkey, from March 2020 to January 2021. We included patients under anticoagulant therapy in the clinical course. The patients were allocated to 3 groups: Group A - low-dose (prophylactic) low-molecular-weight-heparin (LMWH) therapy, Group B - high-dose (therapeutic) LMWH therapy, and patients that received aspirin additional to the high-dose (therapeutic) LMWH as Group C. Primary outcomes were overall mortality rates and length of stay (LOS) in ICU. Secondary outcomes were rates of major hemorrhagic and thrombotic events.

**Results:**

Records of 475 patients were reviewed and 164 patients were included. No significant difference was detected in mortality rates between groups (*p*=0.135). Intensive care unit stay was 13 (9-24.5) days in Group A, 11 (8.75-23) days in Group B, and 13 (9-17) days in Group C without a significant difference (*p*=0.547). No significant difference was detected between groups in terms of thrombotic (*p*=0.565) and hemorrhagic events (*p*=0.615).

**Conclusion:**

A high-dose anticoagulation therapy and addition of aspirin to LMWH therapy did not decrease the mortality rates and LOS in ICU in critical ill COVİD-19 patients. In addition, it did not increase the incidence of major hemorrhage and major thrombotic events.

**S**ince the first days of the coronavirus disease -19 (COVID-19) pandemic, studies have majorly focused on revealing the factors that determine mortality and morbidity of the disease. Also, there has been extensive efforts for conducting research on treatment, but unfortunately to date there is no clinically approved therapeutic drug for COVID-19 disease.^1,2^ Thus, the treatment in patients with COVID-19 have consisted of supportive care, symptomatic treatment such as management of acute pulmonary failure, management of defects in the coagulation system and hemodynamic disorders, management of inflammatory reactions, and interventions against end-organ failures; all of which are mainly carried out in intensive care units (ICUs).^3,4^

The hypercoagulable state that contributes to diffuse microvascular and macrovascular thrombus formations is one of the systematic disorders which may affect the mortality of the patient.^5,6^ Numerous pathogenic mechanisms that may contribute to the hypercoagulability in COVID-19 were reported.^7^ One of the main reasons reported were the hyperinflammatory response mediated by cytokine storm and macrophage activation syndrome, complement activation, and renin angiotensin system overactivation which lead to immune-mediated thrombosis in COVID-19 associated coagulopathy.^7^

There is evidence that support the use of low molecular weight heparin (LMWH) as prophylaxis for venous thromboembolism in critically ill patients.^6^ The published guidelines suggest venous thromboembolism prophylaxis if there is no contraindication for bleeding risk.^8^ Observational studies also support the anticoagulant therapy which may decrease the mortality in the group receiving therapeutic anticoagulation versus patients who do not receive it.^9,10^ Moreover, in the view of the hypercoagulable state, higher-doses of thromboprophylaxis may be crucial in critically ill patients with severe COVID-19 infection. However, the current evidence is limited and the questions of which anticoagulant drug is the most suitable and which doses should be used is still unclear.

According to the guidelines^8^, we administered various regimens of anticoagulant therapy in our institution for critical ill patients during the course of COVID-19 disease. These anticoagulant drugs include a low-dose (thrombophylactic) regimen of LMWH which was primarily administered in the earlier phase of COVID-19 pandemic, high-dose (therapeutic-dose) regimen of LMWH in late phase of the disease and aspirin was used in addition to the LMWH therapy.

We aimed to evaluate the effect of different anticoagulation therapies administered in our institution on the clinical outcomes and rate of ICU-mortality and compare these different regimens in terms of need of invasive mechanical ventilation and length of stay (LOS) in the ICU. Our secondary aim was to reveal the incidences of major hemorrhagic and thrombotic events with the usage of low-dose and higher doses of anticoagulant drugs. Our hypothesis was that the increased doses of LMWH and additional antithrombotic drug usage may lead to a decrease in the morbidity/mortality status of critically ill patients and LOS in ICU.

## Methods

This study was designed as a single-center retrospective case control study and approved by Sakarya University Ethical Board with the approval number 2021-145. The study was carried out according to principles of the Helsinki Declaration. In this study, the researchers followed the Strengthening the Reporting of Observational Studies in Epidemiology (STROBE) reporting guidelines. Medical records of patients who were followed up in the ICU at Sakarya University Hospital, Sakarya, Turkey, between March 2020 and January 2021 were reviewed. Critical ill patients with a positive polymerase chain reaction test for COVID-19 and who received anticoagulant therapy treated with LMWH and aspirin were included in this study. Pregnant and puerperal patients, patients who did not receive anticoagulant therapy, patients followed up in the ICU less than 7 days, patients received intravenous immunoglobulin, interleukin-1 receptor antagonist, and tocilizumab were excluded from the study. The patients were allocated to 3 groups: patients that received low-dose (prophylactic dose) of LMWH per day were included in Group A, patients that received high-dose (therapeutic dose) of LMWH were included in Group B, and patients that received aspirin in addition to LMWH therapy were included in Group C.

We have managed our anticoagulant treatment in critical ill COVID-19 patients according to the guidelines that were established by the National Ministry of Health in November 2020.^11^ These guidelines are based on blood D-dimer levels and recommend routine thromboprophylaxis in all hospitalized COVID-19 patients unless there is active bleeding or thrombocytopenia (<25-30,000/µl). Recommended treatment in prophylaxis is administration of LMWH (enoxaparin 40mg once a day) or heparin (LMWH is preferred). Oral anticoagulants are not routinely recommended for prophylaxis. In patients with high plasma levels of D-dimer, enoxaparin 40 mg 2 times a day is recommended. The use of anticoagulation at the therapeutic dose has also been restricted to limited circumstances. Additionally, administration of 100 mg aspirin in COVID-19 was also advised which was found useful to reduce the pulmonary effect of the disease.^11^

We determined the low-dose (prophylactic dose) LMWH in our study as: 40 mg enoxaparin (or 60 mg according to weight) once a day. High-dose (therapeutic dose) LMWH was determined as: 40 mg enoxaparin (or 60 mg according to weight) 2 times a day. We added 100 mg aspirin in the routine anticoagulant therapy of the critical ill patients after May 2020 according to the National guidelines.

The primary outcome in this study was overall mortality rates measured as cumulative incidence of overall death in ICU within the groups. Length of stay in ICU, necessity of invasive or noninvasive mechanical ventilation were also evaluated.

The rates of major hemorrhagic and thrombotic events were evaluated as secondary outcomes which were all compared between the groups. Major bleeding definitions were adopted from the International Society on Thrombosis and Haemostasis consensus.^12^

The comorbidities and symptoms existed when the patients were admitted to ICU were also evaluated. Need of supplemental oxygen treatment, duration of invasive mechanical ventilation, time to death of patients that died in ICU, and number of days non-invasine mechanical ventilation treatment were also compared between groups.

Laboratory data each focusing on hematologic, inflammatory and renal outcomes were also assessed and compared between groups. Hematocrit, white blood cell, lymphocyte, and thrombocyte counts were determined as the hematologic outcomes. Inflammatory outcomes were serum D-dimer, procalcitonin, ferritin, and C-reactive protein (CRP) values. Serum creatinine and estimated glomerular filtrate rates (eGFR) levels were assessed as the renal outcomes in our cohort. Laboratory values were obtained at 1^st^ and 7^th^ days in the clinical course of the patients in ICU.

### Statistical analysis

Data were analyzed using the Statistical Package for the Social Sciences, version 20 (IBM Corp., Armonk, NY, USA). We expressed categorical variables as numbers and percentages, and continuous variables as median (range). We have made a descriptive analysis of the variables and expressed them as mean ± standard deviation (SD) in normal distribution, and we stated data that distributed abnormally as median of 25^th^-75^th^ percentile (interquartile range). We implemented χ^2^ and the Student’s t-tests for analyzing the categorical and continuous variables. We applied Fisher’s exact test to analyze small samples. We evaluated the differences between 2 groups by using the Student’s t-test when data were normally distributed and we applied the Mann-Whitney-U test when the assumption of normality was not met. A -value of <0.05 was considered significant.

## Results

We have reviewed medical records of 475 patients who were followed up in ICU at Sakarya University Hospital, Sakarya, Turkey, and were diagnosed with COVID-19 disease. A total number of 164 patients were found eligible and enrolled in the study. Allocations to the groups were as follows: 45 patients were added in Group A, 26 patients were added in Group B, and 93 patients were added in Group C.

Demographic characteristics of the patients were comparable in each group including age, gender, and body mass indexes (Table 1). The symptoms of the patients recorded during their admission to the ICU were fever, cough, dyspnea, gastrointestinal symptoms, widespread pain, weakness, and tachypnea. The most common symptom was dyspnea in all groups The second and third most common symptoms were tachypnea and weakness. Tachypnea was significantly more common in Group A (*p*=0.015). There was no significant difference in the comparison of the other symptoms (Table 1).

**Table 1:** - Data of demographic characteristics, comorbidities and symptoms during the admission to the intensive care unit (N=164).

Variables	Group A (n=45)	Group B (n=26)	Group C (n=93)	*P*-values
Age (year), mean±SD	68.64±15.04	66.77±12.88	69.72±11.07	0.564
Gender: male/female (n)	26/19	18/8	68/25	0.191
BMI (kg/m^2^), mean±SD	26.87±5.72	26.56±7.30	27.32±3.37	0.213
***Comorbidities, n (%)***				
COPD	10 (22.2)	6 (23.1)	12 (12.9)	0.266
Coronary disease	5 (11.1)	4 (15.4)	11 (11.8)	0.857
Heart failure	4 (8.9)	4 (15.4)	12 (12.9)	0.687
Diabetes	14 (31.1)	11 (42.3)	23 (24.7)	0.209
Hypertension	18 (40.0)	13 (50.0)	43 (46.2)	0.679
Chronic renal failure	2 (4.4)	2 (7.7)	4 (4.3)	0.871
Malignant neoplasm	3 (6.7)	2 (7.7)	15 (16.1)	0.210
***Symptoms, n (%)***				
Fever	11 (24.4)	1 (3.8)	15 (16.1)	0.780
Cough	12 (26.7)	5 (19.2)	29 (31.2)	0.473
Dyspnea	35 (77.8)	25 (96.2)	78 (83.9)	0.123
GI	2 (4.4)	0 (0)	8 (8.6)	0.223
Widespread pain	4 (8.9)	3 (11.5)	13 (14)	0.689
Weakness	11 (24.4)	10 (38.5)	32 (34.4)	0.385
Tachypnea	33 (73.3)	15 (57.7)	44 (47.3)	0.015*

* *p*<0.05, COPD: chronic obstructive pulmonary disease, BMI: body mass index, GI: gastrointestinal, SD: standard diviation

Hypertension was the most common comorbidity in the cohort. Diabetes was the second, and chronic obstructive pulmonary disease was the third common comorbidity. There was no significant difference between the groups regarding the comorbidities (Table 1).

The methods used to provide supplemental oxygen were oxygen mask with a reservoir, high-flow nasal cannula (HFNC), non-invasive positive pressure ventilation (NIPPV), invasive positive pressure ventilation (IPPV). Among these methods, HFNC (*p*=0.047) and NIPPV (*p*=0.026) were applied significantly higher in patients in the C group than in the other groups (Table 2).

**Table 2:** - Comparison of groups regarding the method of oxygen supplement therapy (N=164).

Oxygen supplement modality	Group A (n=45)	Group B (n=26)	Group C n=93)	*P*-values
Oxygen mask with reservoir	20 (44.4)	9 (34.6)	45 (48.4)	0.457
High-flow nasal cannula	4 (8.9)	1 (3.8)	19 (20.4)	0.047*
NIPPV	3 (6.7)	2 (7.7)	21 (22.6)	0.026*
IPPV	37 (82.2)	24 (92.3)	83 (89.2)	0.371

* *p*<0.05, NIPPV: noninvasive positive pressure ventilation, IPPV: invasive positive pressure ventilation

There was no significant difference between the groups in terms of intensive care hospitalization days (*p*=0.547). The groups were also similar in terms of time until discharge from ICU, number of IPPV days and the duration of time to death. Intensive care follow-up data was expressed in detail in Table 3.

**Table 3:** - Comparison of intensive care follow-up data between groups (N=164).

Intensive care follow-up data	Group A (n=45)	Group B (n=26)	Group C (n=93)	*P*-values
Number of total days in ICU median (IQR)	13 (9-24.5)	11 (8.75-23)	13 (9-17)	0.547
Time until discharge of patients that survived from ICU (days) median (IQR)	14.5 (8.75-33)	23 (13.25-32.75)	12 (7.5-27)	0.692
Number of days IPPV applied, median (IQR)	11 (9-18,5)	9 (5.5-16)	10 (6-15)	0.066
Time until death of patients in ICU (days) median (IQR)	13 (9-23)	10.5 (8-14)	13.5 (9-16.75)	0.381
Hemorrhage requiring transfusion n (%)	9 (20.0)	5 (19.2)	13 (14.0)	0.615
Thrombotic event n (%)	3 (6.7)	4 (15.4)	10 (10.8)	0.565
Mortality rate (%)	77.8	84.6	90.3	0.135

ICU: intensive care unit, IPPV: invasive positive pressure ventilation, IQR: interquartile range

The overall in-ICU mortality rate was 77.8% in group A, 84.6% in group B, and 90.3% in group C. There was no significant difference in mortality rates between the groups (*p*=0.135). There was no significant difference between the groups in terms of thrombotic events (*p*=0.565) and hemorrhagic events requiring transfusion (*p*=0.615; Table 3).

**Figure 1:**
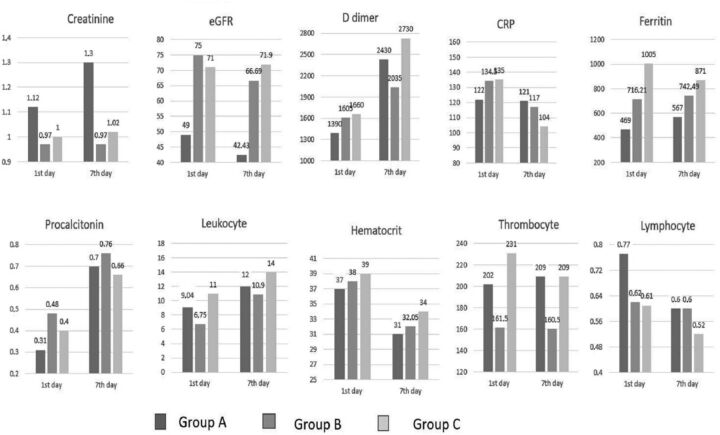
- Laboratory values on the first and seventh days. CRP: C-reactive protein, eGFR: estimated glomerular filtration rate

The comparison of laboratory values between the groups which were obtained at admission to the ICU and on the 7^th^ hospitalization day are shown in Table 4 and Figure 1. Ferritin value was found significantly higher in group C than in group A on the day of admission to the ICU (*p*=0.002). Thrombocyte value was found significantly lower in group B than in group C (*p*=0.003). Leukocyte values on the day of admission and on 7^th^ day of hospitalization in the ICU were found significantly higher in group C than in group B (*p*=0.005), and hematocrit values were found lower in group A than in group C (*p*=0.012). In terms of other laboratory parameters, no significant difference was found between the groups.

**Table 4:** - Data of laboratory tests compared between groups. The values are given as median (IQR) obtained on 1st day / 7th day in intensive care (N=164).

Variables (normal range)	Group A(n=45)	Group B(n=26)	Group C(n=93)	P-values
Creatinine (0.51-0.95 mg/dl)	1.12 (0.74-1.9)/1.3 (0.63-2.3)	0.97 (0.7-1.58)/0,97 (0.62-1.92)	1 (0.71-1.43)/1,02 (0.69-1.88)	0.652/0.725
EGFR (>90 mL/dk)	49 (30.5-87.03)/42,43 (21.04-94.5)	75 (35.15-89.37)/66.69 (31.5-97.42)	71 (42.97-95)/71.9 (30.55-97.02	0.226/0.470
D-dimer (0-500 ugFEU/L)	1390 (629-2820)/2430 (1285-8270)	1605 (1030-2902,5)/2035 (1104-6145)	1660 (708-3700)/2730 (1575-6300)	0.592/0.591
CRP (0-5 mg/L)	122 (44.5-156.5)/121 (68.9-196.5)	134.5 (53.15-180.75)/117 (56.78-168.5)	135 (56.80-190)/104 (51-173)	0.436/0.437
Ferritin (5-204 mcg/L)	469 (169.44-1130)/567 (302.36-1393)	716.21 (246-1476)/742.49 (425-1859)	1005 (490-1844)/871 (578.5-1418)	0.002*/0.064
Procalcitonin (<0.5 ng/ml)	0,31 (0.16-2.04)/0,70 (0.29-3.26)	0,48 (0.29-3.47)/0,76 (0.31-4.13)	0,40 (0.17-1.29)/0,66 (0.25-2.65)	0.408/0.735
Leukocyte(4.6-10.2 K/uL)	9.04 (7-13.5)/12 (8.97-18)	6.75 (4.82-10.46)/10.9 (6-13.25)	11 (7.71-15.15)/14 (10.05-19.6)	0.005*/0.005*
Hematocrit (37.7-53.7%)	37 (33.75-42)/31 (27.2-36)	38 (33.13-43)/32,05 (27.75-36)	39 (35-43)/34 (29.7-39)	0.282/0.012*
Thrombocyte (142-424 K/uL)	202 (154-284.5)/209 (131-265)	161,5 (126-226)/160,50 (115-214)	231 (173-297)/203 (136-276)	0.003*/0.247
Lymphocyte (0.60-3.4 K/uL)	0.77 (0.49-1.21)/0.6 (0.49-1.03)	0.62 (0.49-1.03)/0.6 (0.46-1.32)	0.61 (0.39-0.99)/0.52 (0.29-0.92)	0.149/0.073

* *p*<0.05, EGFR: Estimated glomerular filtration rate, CRP: C reactive protein, IQR: interquartile range. These values have also been expressed in Figure 1.

## Discussion

This study examined the effect of low-dose LMWH, high-dose LMWH and aspirin on clinical outcome, mortality and major hemorrhagic events in patients followed up in ICU with COVID-19, and determined no clinically significant difference between these thromboprophylaxis regimens.

In clinical and postmortem autopsy studies carried out in the early stages of the COVID-19 pandemic, thromboembolic events were reported to be an important factor in mortality and morbidity.^5,13-15^

Although the evaluation of exact prevalence of thromboembolic events in COVID-19 needs further studies, thromboembolic events seemed to be higher in COVID-19 patients than patients followed in the ICU with indications other than COVID-19.^16,17^ Moreover, thrombotic events may occur despite the prophylactic use of LMWH.^18^ For this reason, the idea of employing higher empirical doses of anticoagulation or using more than one anticoagulant drug has arisen.

The anti-inflammatory and antiviral effect of LMWH was also confirmed which makes LMWHs a suitable anticoagulant drug that would have a positive effect on the proinflammatory hypercoagulable state in COVID-19.^19,20^ Aspirin was also suggested as an antiplatelet agent to play a potential role and may have an effect for decreasing thromboembolic complications when administered in combination with heparin and LMWH.^18^ The majority of the studies have compared the prophylactic dose anticoagulant therapy to no anticoagulant usage and supported prophylactic dose anticoagulants.^21-23^ Although there is a common opinion that anticoagulation has a positive effect on morbidity and mortality of COVID-19 patients, there is controversy in the retrospective studies comparing different doses and different anticoagulant agents especially in severely ill patients. Many retrospective studies reported lower rates of mortality when therapeutic-dose anticoagulation was administered compared to either prophylactic-dose anticoagulation or no anticoagulation; on the other hand, there were studies that found no differences in terms of mortality comparing to therapeutic and prophylactic anticoagulation doses.^24-27^

The retrospective study of Meizlich et al^18^ using propensity score matching with a large cohort reported that intermediate dose anticoagulation was associated with a lower incidence of in-hospital death compared to prophylactic dose anticoagulation. They also found that aspirin usage was associated with lower incidence of in-hospital death compared to no antiplatelet therapy. In contrast, the retrospective analysis of Nadkarni et al^24^ could not find a significant difference in mortality. They compared patients with no anticoagulation with patients receiving therapeutic and prophylactic anticoagulation. Prophylactic anticoagulation was associated with lower mortality compared to therapeutic anticoagulation. However, the difference was not statistically significant. A multicenter retrospective study found that aspirin use decreased the risk of mechanical ventilation, ICU admission, and in-hospital mortality without a difference in major bleeding or overt thrombosis between aspirin users and nonusers.^28^ However, the results have been obtained after adjustment of confounding variables; the researchers reported that they could not find a crude association with aspirin usage and in-hospital mortality.

The association of pre-admission antiplatelet/anticoagulant use (aspirin, clopidogrel, warfarin, apixaban, dabigatran, LMWH, and rivaroxaban) at the time of infection with COVID-19 and mortality was also evaluated. The researchers could not find a statistically significant effect on mortality in patients with COVID-19 who were on anticoagulant or antiplatelet therapy due to their previous cardiovascular disease or thrombotic disorders.^29^

All controversial results found in previous studies constituted the necessity of further studies assessing the effect of using different doses and agents of anticoagulant treatment on the clinical course in COVID-19 patients. Randomized controlled prospective studies are very few and majority of them are being continued. Different research groups reported their ongoing studies comparing different anticoagulant agents (such as unfractionated heparin versus LMWH or bivalirudin versus LMWH/UFH) for their effects on mortality and need for mechanical ventilation.^30-32^

Recently, Tang et al^9^ introduced the concept of sepsis-induced coagulopathy (SIC). They stated only patients who meet the SIC criteria or have significantly higher serum D-dimer values can benefit from anticoagulant therapy with LMWH. This may be the reason why the expected decrease in mortality rates is not observed in patients with COVID-19 despite the use of prophylactic and therapeutic doses of anticoagulants or the combined use of antiaggregant and anticoagulant drugs.

A randomized controlled study compared therapeutic anticoagulation (oral rivaroxaban subcutaneous enoxaparin or intravenous unfractionated heparin) to prophylactic anticoagulation (standard in-hospital enoxaparin or unfractionated heparin) for patients with COVID-19.^33^ They found that rivaroxaban or enoxaparin followed by rivaroxaban did not improve clinical outcomes and increased bleeding was observed compared with prophylactic anticoagulation. They concluded that the use of therapeutic dose rivaroxaban should be avoided in these patients. In a recent randomized study by Lamos et al,^34^ they reported an improvement in gas exchange and a decrease in the need for mechanical ventilation when therapeutic enoxaparin was used in patients with severe COVID-19.

Jonmaker et al^35^ compared low, medium, and high doses of LMWH which were determined based on local standardized recommendations, and found that high-dose thromboprophylaxis was associated with a lower risk of death compared with lower doses. The definition of therapeutic dose in our study was similar to medium thromboprophylaxis dosing in the study of Jonmaker et al.^35^ We could not detect a decrease in mortality using therapeutic dose compared to prophylactic dose as well. The results of the INSPIRATION randomized controlled trial was also consistent with findings of our study.^36^ This multicenter study compared intermediate-dose (enoxaparin, 1 mg/kg daily) to a standard dosing of prophylactic anticoagulation (enoxaparin, 40 mg daily) among patients admitted to the ICU with COVID-19. They did not find a significant difference in venous or arterial thrombosis events and mortality within 30 days.^36^ Another randomized controlled study compared therapeutic anticoagulation dose of unfractionated or LMWH to pharmacologic thromboprophylaxis and reported that therapeutic-dose anticoagulation with heparin did not increase the probability of survival to hospital discharge or did not provide higher number of days without cardiovascular or respiratory support.^37^

Usage of therapeutic dose LMWH seems to be safe which did not increase the incidence of major haemorogic events and also did not affect the incidence of thromboembolic events in our study. Our results were consistent with the results of Mattioly et al^38^ in which they confirmed the safety and feasibility of using intermediate dose LMWH in hospitalized COVID-19 patients. In addition, we also did not detect an increase in major haemorogic or thromboembolic events when aspirin was added to the LMWH therapy. In contrast to our expectation, the rate of bleeding requiring blood transfusion (14%) was lowest in group C (LMWH+aspirin) and also the rate of thrombotic event was highest in the group A (prophilactic dose LMWH) (6.7%). However, the differences were not found statistically significant. Although larger prospective randomized studies are needed to further confirm this, we could conclude that the addition of 100 mg aspirin to prophylactic dose LMWH therapy may be safe in COVID-19 patients. On the other hand, although the major bleeding risk was reported as low in COVİD-19 patients, there are studies that affirm the fear of increased hemorrhagic adverse events when therapeutic anticoagulation doses are administered.^39^

### Study limitations

The retrospective design, the monocentric cohort, the small number of patients, and the absence of radiologic evaluation of deep venous thrombosis.

In conclusion, the present study shows that the use of therapeutic dose LMWH and addition of aspirin to the therapeutic LMWH regimen did not reduce the mortality and the need of invasive mechanical ventilation was not decreased in critically ill COVID-19 patients followed up in the ICU. The researchers also could not find a significant difference regarding the length of ICU stay, inflammatory, and renal parameters of the patients. Therapeutic doses of LMWH and addition of aspirin to LMWH therapy did not increase the risk of major bleeding and thrombotic events. The results from further prospective randomized clinical trials are needed to clearly determine the clinical effect of anticoagulant therapy in COVID-19 patients and to set guidelines and recommendations.
